# Antiproliferation and Anti-invasion Activity of Binuclear Copper(II) Complex in BRAFi-Resistant Melanoma Cells

**DOI:** 10.1021/acsptsci.6c00033

**Published:** 2026-08-10

**Authors:** Manoel Oliveira de Moraes Junior, Larissa Anastácio da Costa Carvalho, Yasmin Catherine da Paz Mendonça, Renaira Oliveira da Silva, Cleia Justino Nunes, Keli Lima, João Agostinho Machado-Neto, Tiago Rodrigues, Vito Rebecca, Corine Bertolotto, Ana Maria da Costa Ferreira, Silvya Stuchi Maria-Engler

**Affiliations:** † Department of Clinical and Toxicological Analyses, School of Pharmaceutical Sciences, 28133 University of São Paulo, São Paulo, SP 05508-000, Brazil; ‡ Department of Fundamental Chemistry, Chemistry Institute, University of São Paulo, São Paulo, SP 05508-000, Brazil; § Department of Pharmacology, Institute of Biomedical Sciences, University of São Paulo, São Paulo, SP 05508-000, Brazil; ∥ Center for Natural and Human Sciences, Federal University of ABC, Santo André, SP 09210-580, Brazil; ⊥ Department of Biochemistry and Molecular Biology, Bloomberg School of Public Health, Johns Hopkins University, Baltimore, Maryland 21205, United States; # Centre Méditerranéen de Médecine Moléculaire, Université Côte d’Azur, Nice 06204, France

**Keywords:** copper(II) complexes, melanoma, BRAFi-resistance, cell death mechanisms

## Abstract

Melanoma lethality is driven by high intratumoral heterogeneity, metastatic competence, and phenotypic plasticity, which foster rapid emergence of resistance to targeted therapies such as BRAF inhibitors (BRAFi, e.g. vemurafenib). Copper­(II) complexes have emerged as promising anticancer candidates, yet their activity against BRAFi-resistant melanoma remains poorly defined. Here, we show that the binuclear copper­(II) complex [Cu_2_(apyhist)_2_dpam]­(ClO_4_)_4_ (herein referred to as [Cu­(II)­L]) displays low-micromolar cytotoxicity and antiproliferative activity in parental and BRAFi-resistant melanoma models. Binuclear [Cu­(II)­L] suppresses melanoma migration and invasion and reduces tumor burden and dermal invasion in a reconstructed human skin melanoma model with limited toxicity to normal tissue. Mechanistically, [Cu_2_(apyhist)_2_dpam]­(ClO_4_)_4_ triggers a ROS-dependent stress response that is attenuated by N-acetylcysteine, leading to DNA damage, mitochondrial depolarization and caspase-independent cell death. Molecular profiling indicates engagement of cell cycle arrest and stress-adaptation programs, with signatures suggesting ferroptotic-associated, necroptotic and autophagy-related signaling, pointing to a redox-driven, diverse mechanism rather than a single dominant death pathway. Different modulation of key markers (PARP1, H2AX, MLKL, GPX4, LC3B-II, and p62) supports that its multitarget activity can bypass adaptive resistance mechanisms. Collectively, these findings support binuclear [Cu­(II)­L] as a candidate to target invasive and drug-resistant melanoma phenotypes.

## Graphical abstract

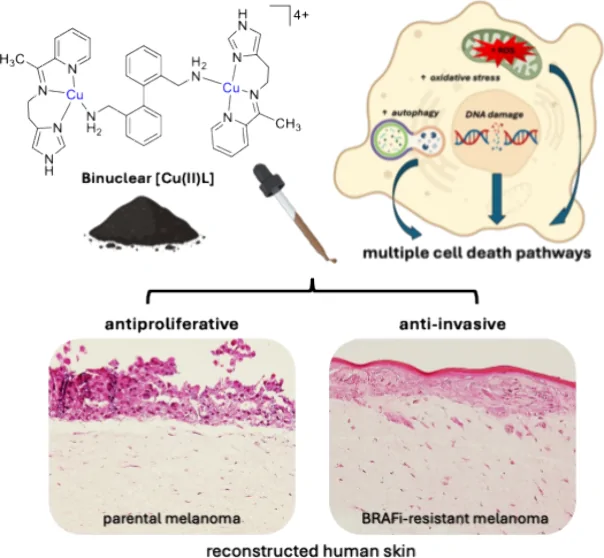

Melanoma is one of the most aggressive forms of skin cancers, defined by its rapid proliferation, strong metastatic potential, and ability to evade the immune surveillance. Although targeted therapies, particularly BRAF inhibitors (BRAFi), have significantly improved both overall survival and progression-free survival, resistance almost invariably develops in many patients.^1,2^ This resistance stems from both intrinsic and acquired tumor heterogeneity, as treatment selects for subpopulations with overexpression or mutations in pathways governing survival, proliferation, metastasis, and resistance to apoptosis.^3^ Identifying new therapeutic strategies to treat BRAFi-resistant melanoma therefore remains an urgent clinical priority.

Copper is a transition metal ion that has attracted significant attention in oncology research.^4–7^ In malignant cells, copper is involved in both direct and indirect regulation of essential processes, including proliferation, angiogenesis, and metastasis.^7,8^ While copper is indispensable for normal cellular metabolism, tumor cells often display altered copper homeostasis, creating a therapeutic vulnerability that can be exploited through controlled delivery strategies.^9,10^

Modulation of intracellular copper­(II) levels in cancer cells has been widely reported to induce potent cytotoxicity through diverse mechanisms, including oxidative stress generation,^11,12^ DNA interaction and cleavage,^13–15^ and activation of regulated cell death pathways such as apoptosis, necroptosis, and ferroptosis.^16–18^

Importantly, copper­(II) complexes embedded in organic scaffolds, especially nitrogenated ligands, offer advantageous pharmacological properties, such as high solubility, lipophilicity, chemical stability, and predictable metabolism.^19^ Because copper also plays a physiological role in basal cellular homeostasis, these complexes may reduce systemic toxicity compared to other metal-based therapies.^20^ Prior studies have shown that mono- and binuclear copper­(II) complexes (Figure 1A) containing a diimine ligand (E)-N-(2-(1H-imidazol-4-yl)­ethyl)-1-(pyridin-2-yl)­ethan-1-imine (apyhist) and bridged by the diamine ligand diphenyl-2,2′-aminomethyl (dpam) exhibit tyrosinase-like activity and potent cytotoxicity against melanoma cells, particularly in models with UVB-induced hyperpigmentation.^21,22^

**Figure 1:**
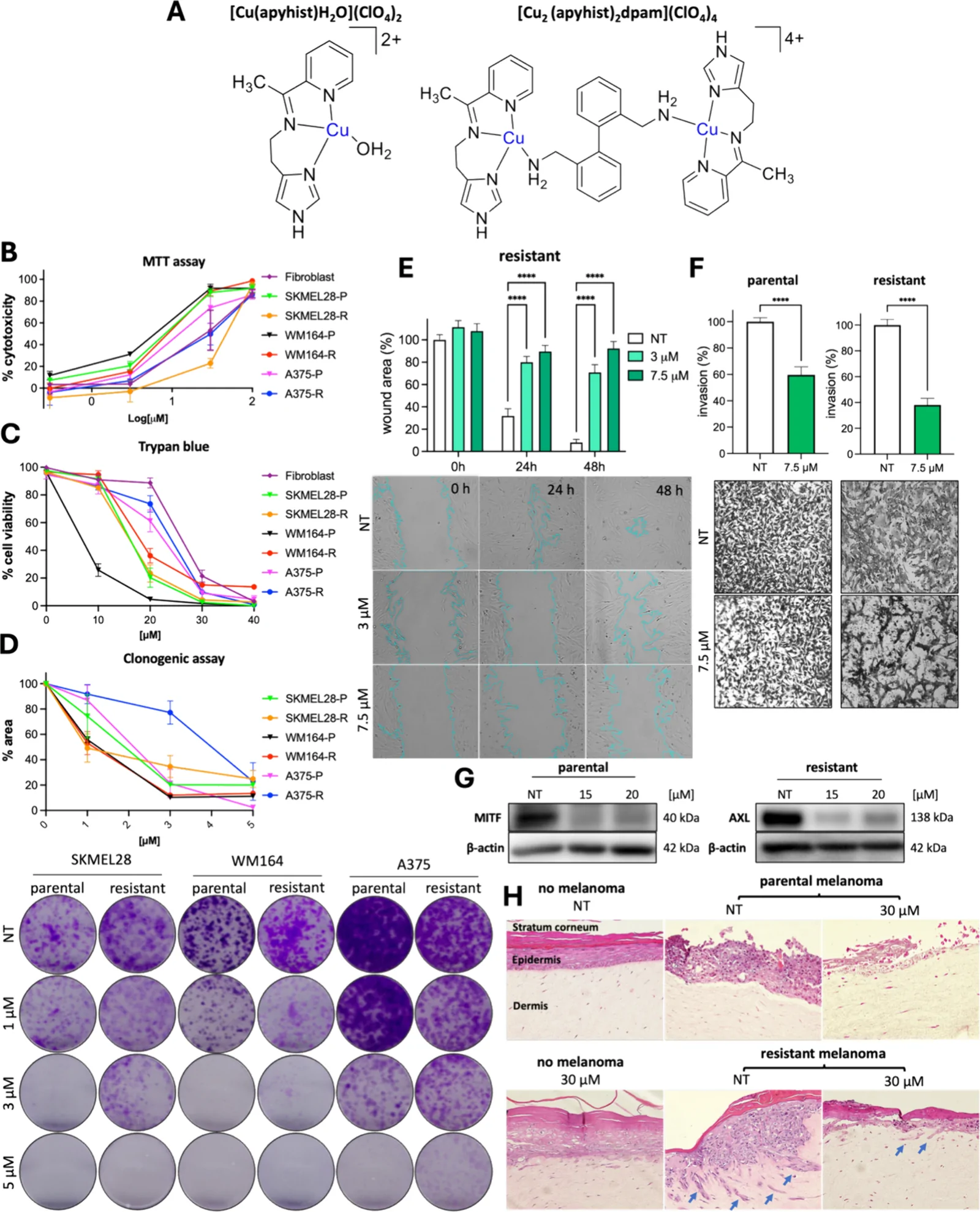
Binuclear copper­(II) complex inhibits proliferation and invasion in parental and resistant melanoma cells. (A) chemical structures of copper­(II) complexes mononuclear [Cu­(apyhist)­H_2_O]­(ClO_4_)_2_ and binuclear copper­(II) complex [Cu_2_(apyhist)_2_dpam]­(ClO_4_)_4_; (B) MTT assay and (C) trypan blue exclusion for 24 h; (D) clonogenic formation assay and representative wells; (E) SKMEL28-R wound-healing migration assay and representative wells; (F) SKMEL28-P/-R invasion assay and representative wells; (G) MITF (SKMEL28-P) and AXL (SKMEL28-R) protein bands after 24-h treatment; (H) hematoxylin and eosin staining of reconstructed human skin with melanoma (SKMEL28). (B–H) All treatments performed with binuclear [Cu­(II)­L] at indicated concentrations. NT = nontreated. Statistical data are expressed as mean ± standard error of the mean (*n* = 3), *p < 0.05, **p < 0.01, ***p < 0.001, ****p < 0.0001. One-Way ANOVA was applied to (E), and Student’s *t* test was used for (F).

Building on this rationale, the present study comprehensively evaluates the effect of the binuclear copper­(II) complex [Cu_2_(apyhist)_2_dpam]­(ClO_4_)_4_, on both parental (-P) and BRAFi-resistant (-R) metastatic melanoma cell lines, with the goal of exploring its cytotoxic potential and mechanisms of action.

## Results

### Binuclear Copper­(II) Complex Exhibits Antiproliferative and Anti-invasive Effects against Parental and BRAFi-Resistant Melanoma Cells

Parental (-P) and BRAFi-resistant (-R) SKMEL28, WM164, and A375 cell lines were treated for 24 h with increasing concentrations of mononuclear and binuclear copper­(II) complexes (Figure 1A), cisplatin, and dacarbazine. Cytotoxicity (growth inhibition) was assessed using the MTT assay, enabling IC_50_ determination (Table 1) and subsequently direct cell viability with the trypan blue exclusion assay (Table 2).

**Table 1:** IC_50_ Values from MTT Assay in 24 h^a^

	WM164-P	WM164-R	SKMEL28-P	SKMEL28-R	A375-P	A375-R	Fibroblasts
mononuclear [Cu(II)L]	>100	>100	>100	>100	>100	>100	ND
binuclear [Cu(II)L]	5.41	8.39	8.18	42.92	14.2	29.21	27.36
	(3.84–7.64)	(6.13–11.47)	(6.06–11.03)	(26.93–68.40)	(7.95–25.34)	(17.47–48.85)	(17.35–43.13)
cisplatin	>100	>100	>100	>100	>100	>100	>100
dacarbazine	>100	>100	>100	>100	>100	>100	>100

a Values are expressed in micromolar (μM) with a 95% confidence interval. ND = not determined.

**Table 2:** IC_50_ Values of Binuclear [Cu­(II)­L] from Trypan Blue Exclusion Assay in 24 h^a^

WM164-P	WM164-R	SKMEL28-P	SKMEL28-R	A375-P	A375-R	Fibroblasts
6.71	18.48	15.92	15.6	22.12	23.09	25.68
(5.25–8.26)	(16.58–20.47)	(14.79–17.77)	(14.62–16.64)	(23.89–24.59)	(21.82–24.4)	(24.55–26.88)

a Values are expressed in micromolar (μM) with a 95% confidence interval.

The binuclear copper­(II) complex displayed low micromolar potency across all melanoma models (Table 1). For WM164, IC_50_ values were 5.4 μM (parental) and 8.4 μM (resistant), representing a modest 1.6-fold reduction in sensitivity. In SKMEL28, resistance was more pronounced, with IC_50_ values increasing from 8.2 μM (parental) to 42.9 μM (resistant cells; 5.2-fold). In A375, IC_50_ rose from 14.2 μM (parental) to 29.2 μM (resistant; 2.1-fold). These findings indicate that, although potency is partially reduced in resistant lines, cytotoxic activity is maintained (Figure 1B).

In contrast, the mononuclear copper­(II) complex, dacarbazine and the metallodrug cisplatin exhibited IC_50_ values above 100 μM, indicating minimal cytotoxic activity in melanoma cells or primary fibroblasts (Table 1; Figure S1, Supporting Information).

Primary fibroblast from healthy donors were affected by the binuclear [Cu­(II)­L] in the trypan blue assay (Figure 1C), with IC_50_ values of 25.7 μM (trypan blue) and 27.36 μM (MTT) (Tables 1 and 2). It is worth mentioning that at 20 μM in trypan blue assay, fibroblast viability decreased by only 12.3% ± 6.6 (Figure 1C), while viability loss exceeded 80% in SKMEL28 and over 90% in parental WM164.

Because of its cytotoxic and antiproliferative activity, the binuclear [Cu­(II)­L] was tested in combination with vemurafenib. At 7.5 μM, it reduced cell viability by 50% in SKMEL28-P (Figure S2, Supporting Information), consistent with prior IC_50_ values (Table 1). Vemurafenib alone reduced viability at 0.03 μM, with around 50% loss at 0.1 μM and around 90% at 3 μM. On the other hand, the combined treatment did not show significant difference around vemurafenib’s IC_50_ suggesting no additive antiproliferative effect.

Clonogenic assays further confirmed activity. WM164-P, WM164-R, and SKMEL28-R cells exhibited strong sensitivity to 1 μM treatment, while SKMEL28-P, A375-P, and A375-R were significantly inhibited at 3 μM, concentrations lower than those needed to affect overall cell viability (Figure 1D).

Given that SKMEL-28 cells showed the lowest and equipotent IC_50_ (15 μM) values across both parental and resistant lines in trypan blue assay (Table 2), they were chosen for subsequent mechanistic investigations. Binuclear [Cu­(II)­L] (15 and 30 μM) induced dose-dependent morphological changes in melanoma cells, shifting from a spread and elongated state to a rounded cytoplasm and detached phenotype. These observations are indicative of cytotoxicity and align with the observed decrease in cell viability (Figure S3, Supporting Information).

Since high MAPK constitutive activity confers metastatic profile to BRAFi-resistant cells,^23^ we tested whether binuclear [Cu­(II)­L] could affect cell mobility. In wound-healing assay, binuclear [Cu­(II)­L] complex inhibited SKMEL28-R migration by 30% at 3 μM and 40% at 7.5 μM after 24 h (Figure 1E). At 48 h, untreated cells had closed 90% of the wound, while treated groups remained at 70% (3 μM) and 10% (7.5 μM), showing significant antimigratory activity (*p* < 0.0001). These results highlight the antimigratory capacity of the binuclear copper­(II) complex at low cell death induction concentrations against the highly migratory BRAFi-resistant cell line.

Boyden chamber assays confirmed anti-invasive effects. At 7.5 μM, the binuclear [Cu­(II)­L] markedly reduced the number of cells traversing the Matrigel barrier in both parental and resistant SKMEL28 cells (Figure 1F). Importantly, these effects occurred at subcytotoxic concentrations, indicating direct anti-invasive activity (Table 2, Figure 1C), underscoring its potential to suppress invasion independently of BRAFi-resistance status. Molecular markers corroborated these effects: MITF (linked to proliferation) and AXL (associated with invasiveness) were both downregulated at 15 and 20 μM (Figure 1G).

**Figure 2:**
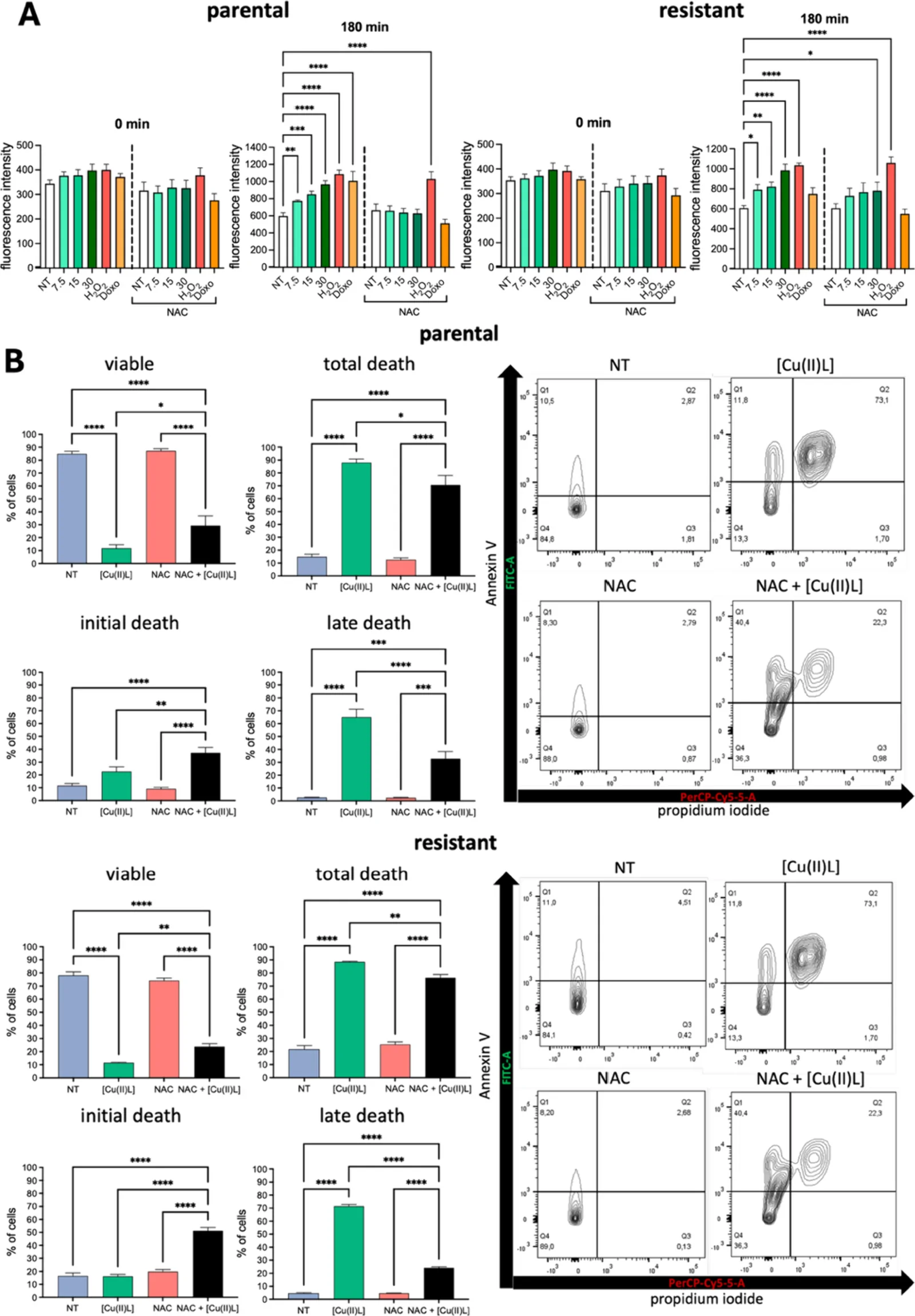
Binuclear copper­(II) complex induces ROS production and blocking oxidative stress inhibits cell death. (A) ROS kinetics with DHR-123 probe and coexposure to NAC (1 mM) for 30 min; (B) cell death evaluation with Annexin V and PI and coexposure to NAC (10 mM) and binuclear [Cu­(II)­L] (20 μM) for 24 h. (A-B) Assays performed in SKMEL28 cells. NT = nontreated; Doxo = doxorubicin (5 μM); NAC = N-acetylcysteine; viable = Annexin V-/PI-, initial death = Annexin V+/PI-, late death = Annexin V+/PI+, total death = sum of Annexin V+/PI-, Annexin V+/PI+, and Annexin V-/PI+ cell populations. Statistical data are expressed as mean ± standard error of the mean (*n* = 3), *p < 0.05, **p < 0.01, ***p < 0.001, ****p < 0.0001; Two-way ANOVA was applied to (A), and One-way ANOVA to (B).

In reconstructed human skin (RHS) 3D model, which recapitulates the structural organization and barrier function of native skin,^24^ the compound exhibited low toxicity in nonmalignant tissue. Untreated RHS with no melanoma displayed intact dermis, epidermis, and stratum corneum layers (Figure 1H). Treatment caused no visible disruption in normal tissue. In melanoma-bearing RHS, parental cells proliferated within the epidermis, while resistant cells invaded dermal layers. Treatment with the binuclear complex markedly reduced both epidermal tumor burden in parental melanoma-bearing RHS and dermal invasion in resistant melanoma-bearing RHS (Figure 1H), confirming anti-invasive and cytotoxic activity in a physiologically relevant model.

### Binuclear Copper­(II) Complex Induces Oxidative Stress, DNA Damage and Mitochondrial Membrane Depolarization

Given that copper­(II) complexes were previously reported to produce intracellular reactive oxygen species (ROS) in cancer cells, we assessed ROS abundance. The binuclear [Cu­(II)­L] induced ROS production in a dose-dependent manner, beginning at 7.5 μM and peaking at 30 μM (Figure 2A). Doxorubicin and H_2_O_2_ were employed as positive controls, inducing a significant increase in ROS levels in melanoma cells as expected. Co-treatment with the antioxidant NAC effectively abrogated ROS induction, supporting the involvement of oxidative stress in the compound’s activity. However, rescue effect may reflect a combination of direct antioxidant action and potential interference with the compound’s metallic coordination chemistry.

We next assessed whether the antioxidant could prevent binuclear [Cu­(II)­L]-induced cell death after 24-h treatment. Thus, cotreatment with NAC significantly reduced the overall population in total death (sum of cells stained as Annexin V+/PI-, Annexin V+/P+, and Annexin V-/PI+) upon binuclear [Cu­(II)­L] (20 μM) treatment in both the parental and resistant lines (Figure 2B). Moreover, NAC markedly decreased the proportion of cells in late death (Annexin V+/P+) and increased the proportion in initial death (Annexin V+/PI-), thereby preserving plasma membrane integrity.

Mitochondrial depolarization measured using tetramethylrhodamine methyl ester (TMRM) was significant at 30 μM in both parental and resistant cells, comparable to positive control carbonyl cyanide m-chlorophenyl hydrazone (CCCP, Figure 3A).

**Figure 3:**
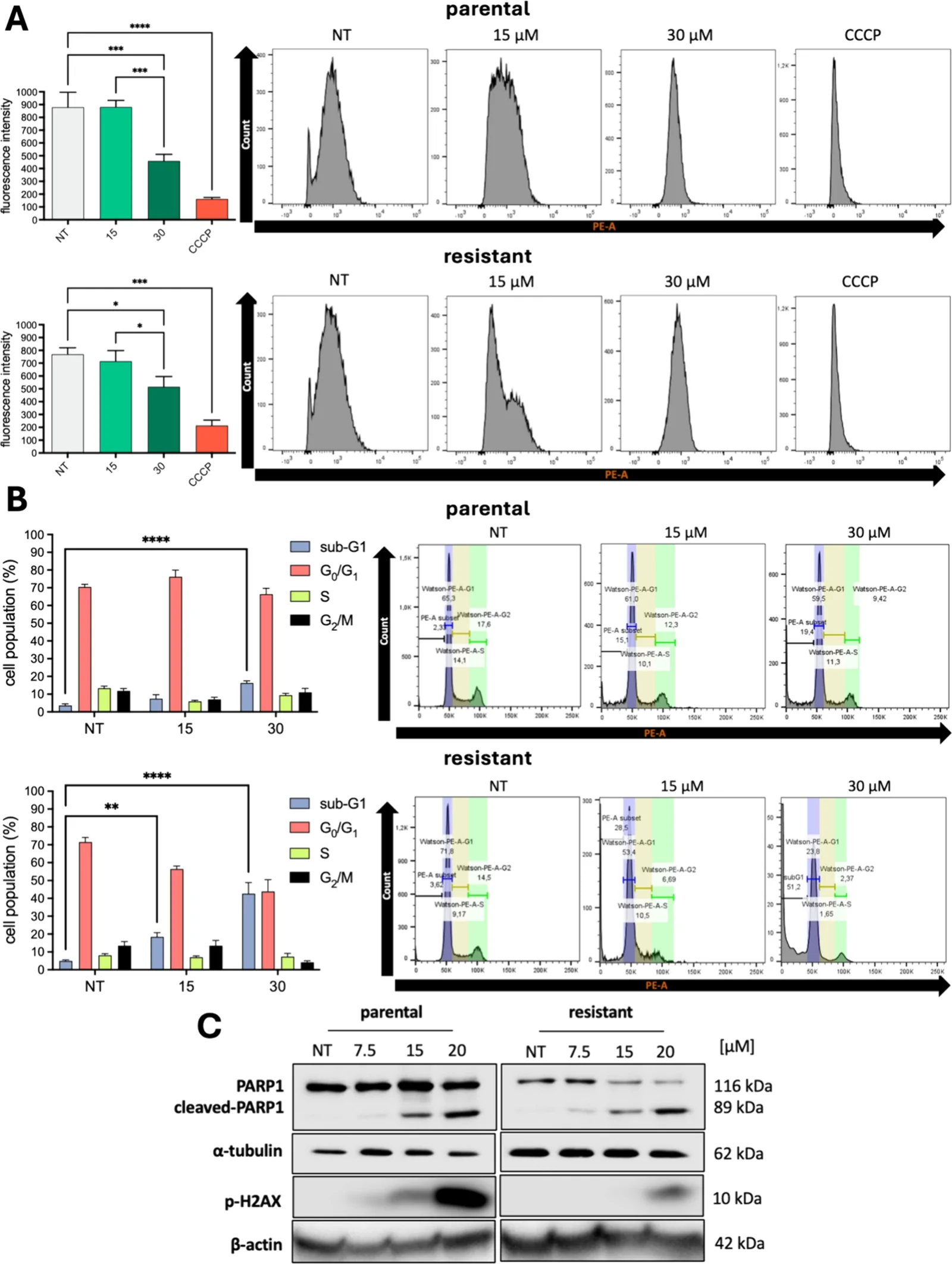
Binuclear copper­(II) complex induces DNA damage-related cell cycle arrest and mitochondrial membrane depolarization. (A) mitochondrial membrane depolarization upon binuclear [Cu­(II)­L] treatment; (B) cell cycle evaluation; and (C) DNA damage markers PARP1 and p-H2AX protein bands. (A-C) All tested with 24-h incubation upon binuclear [Cu­(II)­L] treatment in SKMEL28 cells. NT = nontreated; CCCP = carbonyl cyanide m-chlorophenyl hydrazone (50 μM). Statistical data are expressed as mean ± standard error of the mean (*n* = 3), *p < 0.05, **p < 0.01, ***p < 0.001, ****p < 0.0001; One-way ANOVA was applied to (A), and Two-way ANOVA to (B).

Cell cycle analysis showed an increase in the sub-G1 fraction, consistent with DNA fragmentation. SKMEL28-P cells exhibited increases of 8.2% (15 μM) and 10.1% (30 μM), while SKMEL28-R cells increased by 17.5% and 40.9%, respectively (Figure 3B). Western blot revealed increased PARP1 cleavage and H2AX phosphorylation, both markers of DNA damage by 15 and 20 μM binuclear [Cu­(II)­L] activity, with a simultaneous decrease in the uncleaved PARP1 band relative to the NT control in parental melanoma. In resistant melanoma, cleaved PARP1 bands were already visible at 7.5 μM, with a reduction in total PARP1 bands from 15 μM onward (Figure 3C).

### Binuclear Copper­(II) Complex Activates Multiple Cell Death-Associated Pathways: Necroptosis, Ferroptosis, and Autophagy

To further elucidate the mechanism of action of the binuclear [Cu­(II)­L] complex, a qPCR array comprising genes involved in cell cycle progression and arrest, cell death, apoptosis, and autophagy was performed.

In parental cells, 16 genes were significantly altered. Four genes directly linked to cell cycle progression were downregulated (CCNA2, CCNB1, BIRC5, and CCNE), whereas genes associated with cell cycle arrest were upregulated (CDKN1B/p27, GADD45A, and CDKN1A/p21), along with apoptosis-related genes, (BAK1, BCL2L11, BBC3, and PMAIP1) and autophagy-related genes (ATG5, SQSTM1/p62, and MAP1LC3B) (*p* < 0.05; Figure 4A).

**Figure 4:**
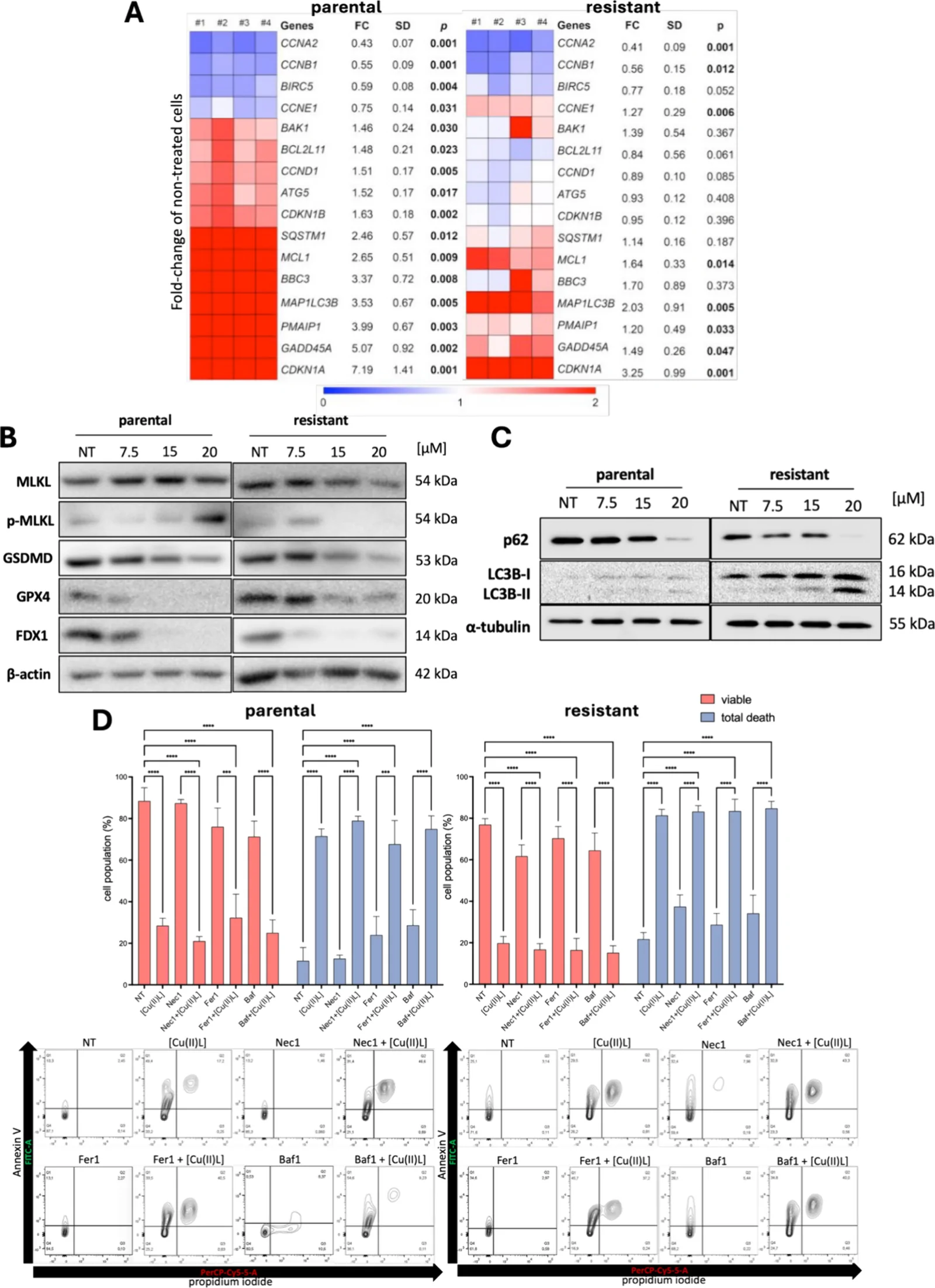
Binuclear copper­(II) complex activates multiple cell death pathways. (A) heat-map gene evaluation, which each colored square represent one biological replicate (*n* = 4) from fold-change of vehicle-treated cells, and downregulated/upregulated genes are shown in blue/red, respectively; (B) types of cell death pathway markers bands; (C) autophagy markers pathway protein bands; (D) cell death evaluation with Annexin V and PI with programmed cell death inhibitors. (A-D) All tested with 24-h incubation with binuclear [Cu­(II)­L] (20 μM) treatment in SKMEL28 cells. NT = nontreated; Nec1 = necrostatin-1 (30 μM); Fer1 = ferrostatin-1 (5 μM); Baf = bafilomycin A1 (0.01 μM); viable = Annexin V-/PI-; total death = sum of Annexin V+/PI-, Annexin V+/P+, and Annexin V-/PI+ cell populations; FC = fold-change; SD = standard-deviation. Statistical data are expressed as mean ± standard error of the mean (*n* = 3), *p < 0.05, **p < 0.01, ***p < 0.001, ****p < 0.0001; Two-way ANOVA was applied to (B).

In BRAFi-resistant melanoma, 8 genes were modulated again favoring cell cycle arrest and autophagy induction with downregulation of CCNA2 and CCNB1, and increased expression of GADD45A, CDKN1A/p21, MCL1, PMAIP1, and MAP1LC3B (LC3B).

These findings indicate that BRAFi-resistant cells exhibited a lower degree of gene modulation compared to parental cells. The antiproliferative machinery was strongly engaged overall, particularly in parental cells and to a lesser extent in resistant cells. Another notable finding was the increased expression of autophagic machinery, represented by MAP1LC3B (LC3B), in both parental and resistant cells.

Further, Annexin V and PI staining evidenced that caspase inhibition (z-VAD-fmk) failed to rescue viability and decrease overall cell death in parental and resistant melanoma cells (Figure S4A, Supporting Information). Western blot showed not only an inability to activate caspases via their cleavage in both parental and resistant melanomas but also a reduction in total caspase-3 and caspase-9 bands as the concentration of the binuclear [Cu­(II)­L] complex increased in resistant melanoma cells (Figure S4B, Supporting Information). Altogether these results indicate that the binuclear copper­(II) complex triggers a caspase-independent cell death program.

Given the antiproliferative effects of the binuclear [Cu­(II)­L] in parental and resistant melanoma cells, we next sought to define the specific cell death programs engaged by this molecule considering the cytotoxic mechanisms described above. We therefore analyzed key proteins associated with distinct death pathways in parental and resistant SKMEL28 cells focusing on necroptosis (MLKL and p-MLKL), pyroptosis (GSDMD), ferroptosis (GPX4), and cuproptosis (FDX1) markers (Figure 4B).

GSDMD and FDX1 levels decreased in a concentration-dependent manner, with a more pronounced reduction in resistant cells, arguing against pyroptosis or cuproptosis as major contributors to cytotoxicity under these conditions.

GPX4 was also decreased in [Cu­(II)­L] treated group relative to the NT control, consistent with impaired detoxification of lipid peroxides, aligning with ferroptosis-associated signaling in the activity of the binuclear [Cu­(II)­L].^25^

In parental cells, MLKL phosphorylation increased at 20 μM while total MLKL remained unchanged, indicating activation of necroptosis signaling. In contrast, resistant cells exhibited depletion of basal phospho-MLKL beginning at 15 μM together with a reduction in total MLKL, suggesting that necroptosis is not engaged in the BRAFi-resistant context.

Given prior evidence of autophagy induction by related copper complexes^22^ and our gene expression data, we also evaluated autophagy markers (LC3 and p62). In parental cells, the compound modestly increased LC3 lipidation (LC3B-II), and reduced p62 (Figure 4C). In resistant cells, autophagy features were more pronounced; LC3B-II accumulation was evident at 15 μM and particularly at 20 μM, accompanied by decreased p62 at both concentrations.

In this context, to elucidate the role of these signaling death pathways, cells were treated with the binuclear [Cu­(II)­L] (20 μM) in combination with inhibitors of necroptosis (necrostatin-1), ferroptosis (ferrostatin-1), and autophagy (bafilomycin A1) employing Annexin V and PI as markers of cell death. None of the inhibitors was able to significantly rescue cell viability following treatment with the binuclear [Cu­(II)­L], as cotreatment groups exhibited death levels comparable to those observed with [Cu­(II)­L] single agent (Figure 4D). Taken together, these results show binuclear [Cu­(II)­L] complex induced cytotoxicity, DNA damage, and mitochondrial dysfunction in an oxidative stress-dependent manner.

## Discussion

Collectively, our findings demonstrate that the binuclear [Cu­(II)­L] exerts a broad spectrum of antitumor effects against metastatic melanoma, impacting both parental and BRAFi-resistant cells. These effects were consistently observed across complementary platforms, including two-dimensional assays, three-dimensional reconstructed human skin (RHS) models, and molecular profiling. The compound inhibited proliferation, suppressed migration and invasion, and engaged stress- and regulated cell-death-related programs. Mechanistically, binuclear [Cu­(II)­L] induced oxidative-stress-dependent cytotoxicity, DNA damage, and mitochondrial dysfunction.

Changes in key markers (PARP1, H2AX, MLKL, GPX4, LC3B-II, and p62) were consistent with the involvement of multiple downstream death-related modules; however, the lack of rescue by pathway-specific inhibitors indicates a redox-driven, multitarget response with redundancy across executors rather than dependence on a single dominant regulated cell-death pathway. Distinct modulation of key markers (PARP1, p-H2AX, MLKL, GPX4, LC3B-II, and p62) supports that binuclear [Cu­(II)­L] multitargeting bypasses adaptive resistance mechanisms.

Copper­(II) complexes have attracted growing interest in oncology due to their ability to interfere with cancer cell metabolism, redox homeostasis, and survival signaling.^5^ They can trigger diverse programmed cell death pathways, cuproptosis, necroptosis, ferroptosis, and autophagy-dependent cell death^11–18^ that might depend on the cellular context.

By exploiting the elevated copper requirements of tumor cells, these compounds can achieve preferential cytotoxicity, although the sensitivity window varies relative to the specific malignancy and resistance profile of the target cells.^26–30^ The structural versatility further allows pharmacokinetic and pharmacodynamic optimization.^7^

Our MTT assay showed stronger antiproliferative effects in parental compared to resistant cells, consistent with the reduced baseline proliferation typical of resistant phenotypes.^23^ However, trypan blue assays and clonogenic assays revealed that both parental and resistant cells remained vulnerable to membrane integrity loss and long-term growth suppression, underscoring the therapeutic potential of binuclear [Cu­(II)­L].

Importantly, a limitation of the current study is that Annexin V/PI-based flow cytometry was not performed on primary fibroblasts. Consequently, while overall metabolic decline and viability loss were confirmed by MTT and trypan blue assays, the precise cell death pathways triggered by binuclear [Cu­(II)­L] in nonmalignant cells remain to be fully characterized.

Given the role of the MAPK pathway in melanoma invasiveness,^3,31^ it is noteworthy that the binuclear [Cu­(II)­L] complex inhibited migration and invasion in both parental and resistant SKMEL28 cell lines, as supported by functional assays and the RHS model.^23,32^ At higher concentrations, the compound also reduced AXL levels, consistent with suppression of an invasive program. Importantly, this occurred at concentrations below overt cytotoxic thresholds, suggesting direct antimetastatic activity.

Mechanistically, the complex increased oxidative stress, DNA damage, mitochondrial dysfunction, sub-G1 populations and PARP1 cleavage with H2AX phosphorylation confirmed DNA-damage mediated cytotoxicity. ROS induction and its attenuation by NAC, together with NAC-mediated reduction of cell death, support a central role for oxidative stress in the cytotoxic cascade; however, because NAC can influence metal availability/chemistry, additional antioxidant strategies with distinct mechanisms will be valuable to further strengthen ROS causality. In addition, our group has previously reported that compounds triggering oxidative stress, DNA damage (with H2AX activation), and endoplasmic reticulum stress possess potent cytotoxicity against melanoma cells resistant to BRAF inhibition.^33,34^

Further, GADD45A and PMAIP1 gene expression was increased in both parental and resistant cells; as these genes are associated with cell death and cell cycle arrest in response to DNA damage,^35,36^ and their downregulation can promote proliferation and motility in cancer cells.^37^ Also, previous results have already demonstrated the ability of binuclear [Cu­(II)­L] to induce DNA double-strand cleavage outside the cell.^22^

Although PARP1 induction is not a classical apoptosis marker and may be involved in other types of cell death via DNA damage,^38^ the activation of caspase 3 and 7 is closely linked to PARP1 cleavage and consequently apoptosis; as well as caspase 3 and 9 are related to apoptosis via mitochondria.^39–41^ Notably, PARP1 processing can also occur through noncaspase proteases under intense oxidative stress and DNA damage, which may explain the dissociation between PARP1 cleavage and the lack of detectable caspase activation in our system. However, caspase activation was absent, and z-VAD-fmk failed to block cell death, indicating caspase-independent pathways.

Our analyses revealed necroptosis in parental cells (MLKL phosphorylation), ferroptosis-associated signaling in both parental and resistant cells (GPX4 depletion), and strong autophagic features, especially in resistant cells (LC3B-II accumulation and p62 reduction). Conversely, BRAFi-resistant melanoma cells showed decreased total and phosphorylated MLKL. These results point to a difference in the mechanism of action and cellular susceptibility between parental and BRAFi-resistant melanoma, suggesting that necroptosis may be important in parental cells but blocked in resistant cells.

Although ferroptosis was classically described as a process exclusively induced by iron,^42^ recent evidence suggests that copper can also promote ferroptosis under certain conditions, disrupting mitochondrial homeostasis and inducing oxidative stress, ultimately leading to ferroptosis in cancer cells.^43–45^ In this context, GPX4 depletion may reflect impaired lipid peroxide detoxification, contributing to a pro-ferroptotic environment that likely arises from a combination of mixed direct and secondary effects of the binuclear [Cu­(II)­L].

Therefore, despite the observed downregulation of GPX4, we acknowledge certain limitations in definitively characterizing the cell death mechanism as canonical ferroptosis. The absence of rescue effects by the specific inhibitor ferrostatin-1 suggests that while ferroptotic signaling components are engaged, they do not represent the sole or dominant execution pathway. Consequently, our findings point toward a complex, ROS-driven death phenotype where multiple signaling signatures coexist, in which ferroptotic-associated markers play a contributory rather than a defining role.

Furthermore, these results confirm that the binuclear copper­(II) complex induced an autophagy-related process, evidenced by increased LC3 activation and the presence of acidic vesicular organelles in melanoma cells,^22^ and also indicate greater autophagic activity in resistant cells compared with parental cells, potentially leading to autophagic cell death or representing an attempt by the cells to survive the cytotoxic action of this molecule. Given the absence of viability rescue with autophagy inhibition, our data support that autophagy is robustly engaged, particularly in resistant cells, but may act as a stress-adaptation module rather than a single decisive execution route.

Regardless of this consequence, the differences in the autophagic response to binuclear [Cu­(II)­L] between resistant and parental cells align with the nature of BRAFi-resistance mechanisms. As demonstrated by several studies, resistant cells employ autophagy induction as one of their primary defense mechanisms against cell death pathways.^3,46^ In this context, the binuclear [Cu­(II)­L] may overstimulate autophagy to a lethal threshold, effectively converting it into a death pathway. Alternatively, sustained autophagy may reflect a compensatory response to oxidative and mitochondrial stress that becomes insufficient as damage accumulates.

Although autophagy inhibition strategies, aimed at facilitating apoptosis induction, have been studied and successfully applied by anticancer molecules in resistant cells,^47,48^ exacerbated induction of autophagy in cells where autophagy plays a key survival role has proven to be an ally in overcoming this resistance phenotype.^49–51^

The synergistic combination of multiple molecules to induce multiple death pathways has proven relevant, as certain cancer types may develop resistance to specific cell death pathways.^34,52–54^ Notably, clinically approved anticancer drugs can induce different types of cell death depending on the cell type, as seen with paclitaxel, which can trigger apoptosis, necroptosis, pyroptosis, ferroptosis, and autophagy under different contexts.^55,56^ There are also examples of single molecules capable of inducing multiple mechanisms of action with different types of cell death within the same cell.^57–63^

Although the lack of selectivity and multitarget mechanisms of action may raise concerns regarding potential adverse effects and off-target interactions,^64^ our reconstructed human skin data indicate a favorable therapeutic index, with reduced toxicity in nonmalignant tissues. Moreover, strategies that simultaneously activate multiple cell death programs may help overcome resistance that emerges when tumors adapt to a single pathway.^52,65,66^

In the past decade, several research groups have invested in the design, synthesis, and cytotoxic characterization of various copper complexes with potential anticancer activity. Despite the challenges of developing a new drug, especially a metal-based complex, copper­(II) complexes have shown promise in this regard, with a significant portion of molecules exhibiting similar mechanisms of action, triggering multiple types of programmed cell death through increased oxidative stress, DNA damage, and mitochondrial activity impairment,^11–18,67–69^ which appears to be the case for our binuclear copper­(II) complex.

## Conclusion

In summary, our study demonstrates that a binuclear copper­(II) complex inhibits melanoma cell proliferation and invasion while engaging multiple stress and regulated cell-death-related programs, in which induced oxidative stress seems to play an essential role. In parental cells, marker changes suggest engagement of necroptosis-, ferroptosis-, and autophagy-related signaling, whereas BRAFi-resistant cells show a profile dominated by ferroptosis-associated and autophagy-related pathways with reduced MLKL phosphorylation (Figure 5). Since none of the pathway-specific inhibitors restored viability, these programs likely represent parallel or secondary executors within a broader redox-driven cytotoxic mechanism rather than exclusive routes. These findings highlight the therapeutic promise of copper­(II)-based compounds as novel strategies to circumvent BRAFi-resistance in advanced melanoma.

**Figure 5:**
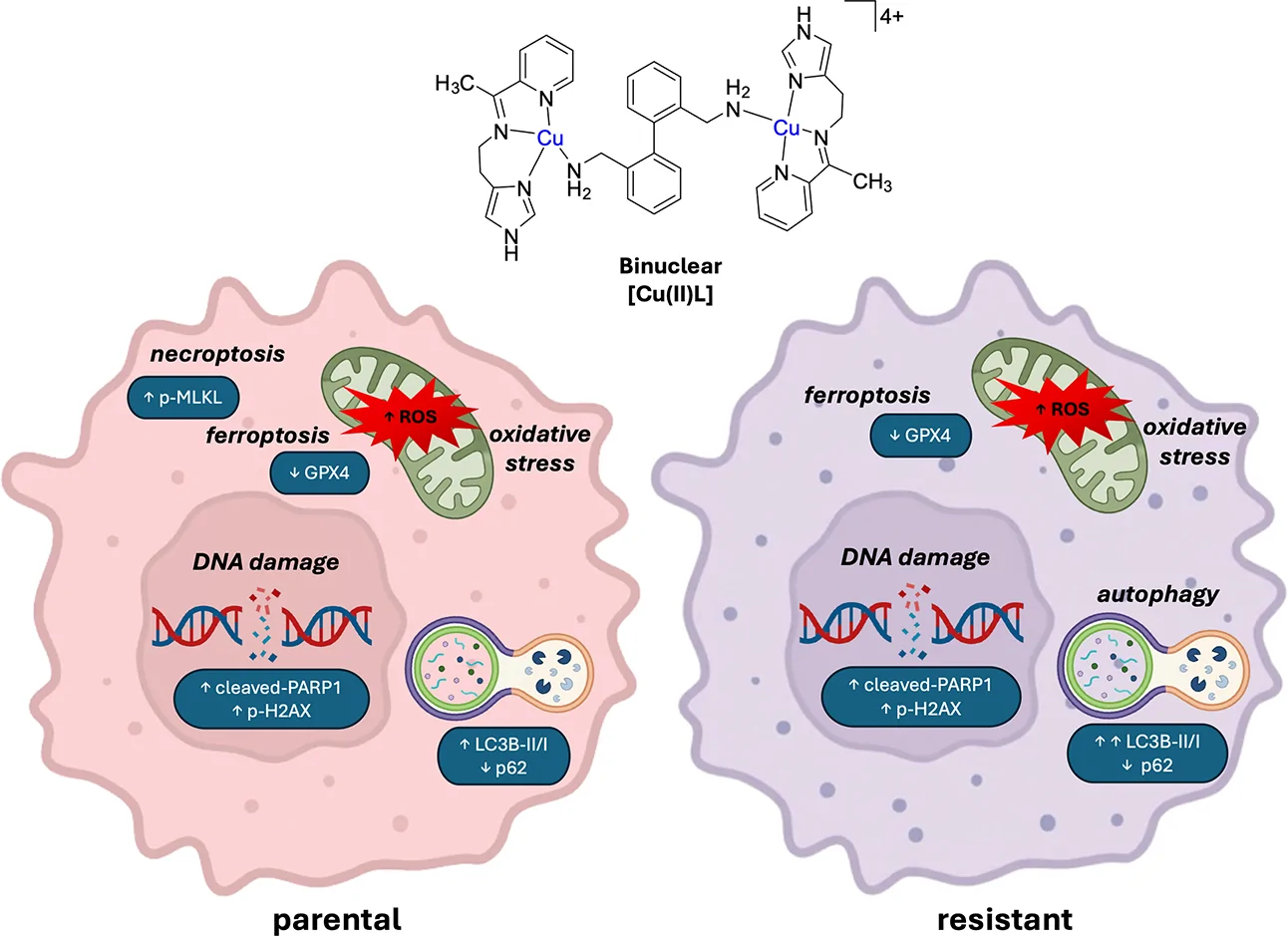
Proposed mechanism of action as an effect of binuclear [Cu­(II)­L] on parental and resistant melanoma cells. ↑ = upregulation; ↑↑ higher upregulation; ↓ downregulation; ROS = reactive oxygen species. Generated with Biorender.com.

## Methods

### Syntheses of complexes [Cu­(apyhist)­H_2_O]­(ClO_4_)_2_ and [Cu_2_(apyhist)_2_dpam]­(ClO_4_)_4_

The mononuclear complex has been synthesized, as previously reported,^21^ by condensation reaction of 2-acetylpyridine with histamine, in ethanol–water solution, forming the consequent imine, followed by metalation in situ with copper­(II) perchlorate hexahydrate (98%). The formed complex was isolated as green crystals, at pH 7.5.

Calc. for C_12_H_16_N_4_CuCl_2_O_9_ (FW = 494.73 g/mol): C, 29.14; H, 3.26; N, 11.33; Cu, 12.83%; Experimental values: C, 29.26; H, 3.50; N, 11.30; Cu, 12.63%. UV/vis bands, λmax, nm (ε, 103 M-1 cm-1): 208 (31.5), 277 s (4.1), 285 (8.0), 295 s (5.8), 632 (0.084).

For the binuclear complex, the precursor mononuclear complex was prepared as described above, followed by the addition of stoichiometric amounts of the bridge-ligand 2,2′-bis­(aminomethyl)­diphenyl (dpam). Thereafter, brownish-violet crystals of the resulting binuclear species were collected, washed with cold methanol, and dried in a desiccator under low pressure.

Calc. for C_38_H_44_N_10_Cu_2_Cl_4_O_16_ (FW = 1165.72 g/mol): C, 39.15; H, 3.80; N, 12.02; Cu, 10.90%; Experimental values: C, 39.45; H, 3.83; N, 11.48; Cu, 11.09%. UV/vis bands, λmax, nm (ε, 103 M-1 cm-1): 277 s (16.0), 285 (18.0), 295 s (14.0), 510 (0.48), 664 (0.175).

Characterization of both compounds was provided by UV/vis and EPR spectroscopies, in addition to elemental analyses, as described elsewhere.^21^

### Experimental model and cell culture

Human immortalized cell lines were used as melanoma models, namely SKMEL28 BRAF^V600E^ (ATCC HTB-72), WM164 (CVCL_7928) BRAF^V600E^, and A375 BRAF^V600E^ (CRL_1619), referred to as parental lines. From these cell lines, BRAFi-acquired resistance phenotypes (-R) were developed using increasing concentrations of the BRAFi, vemurafenib, (PLX4032, SelleckChem, catalog no. S1267) as previously described by our group.^70^ BRAFi-resistant cells were cultured continuously in the presence of vemurafenib at the following concentrations: SKMEL28-R at 3 μM; A375-R and WM164-R at 4.5 μM. Cells were cultured in DMEM (Gibco, catalog no. 12100) with 10% fetal bovine serum (FBS), 25 μg/mL ampicillin (Merck, catalog no. PHR1214), and 100 μg/mL streptomycin (Gibco, catalog no. 11860) at 37 °C with 5% CO_2_ in a humidified chamber.

Human samples such as primary fibroblasts, keratinocytes and melanocytes were isolated from skin obtained from breast plastic surgeries and/or posthectomies. All human sample experiments were performed following the protocols evaluated and approved by the University Hospital (Ethics Approval Number CEP/HU-USP 943/09, SISNEP CAAE 0062.0.198.000-9) and Hospital das Clínicas of the School of Medicine, University of São Paulo (Ethics Approval Number CAAE: 0089.6.612.4.0000.0068), in accordance with approval from the local ethics committee (Ethics Approval Number CEP/FCF-USP 534).

### Western Blot

Cells were lysed with RIPA buffer (10 mM TRIS pH 7.5, 150 mM NaCl, 1% NP40, 0.1% SDS, 1% sodium deoxycholate, 5 mM EDTA, 5 mM EGTA, 25 mM NaF, and 1 mM Na_2_VO_4_) supplemented with protease inhibitors. Subsequently, 30 μg of total protein was quantified from the cell lysed supernatant. Protein-loaded samples were separated by electrophoresis 4–20% Mini-PROTEAN TGX (BioRad, catalog no. #4561096) and transferred to a polyvinylidene difluoride (PVDF) membrane, which was blocked using low-fat 5% milk for 1 h. Membranes were incubated with the primary antibody overnight at 4 °C and secondary antibody for 1 h at room temperature (Table 3). Detection was performed using an enhanced chemiluminescence system (Millipore, catalog no. WBLUF0500), and densitometry was analyzed using the Fiji 2.16 software.

**Table 3:** Antibodies List

Antibody	Manufacturer	Catalog no.
Caspase 3	Cell Signaling	#9662
Caspase 9	Cell Signaling	#9502
MITF	Abcam	Ab12039
AXL	Cell Signaling	#8661
PARP1	Cell Signaling	#9542
LC3B	Cell Signaling	#2775
SQSTM1/p62	Cell Signaling	#88588
p-H2AX	Cell Signaling	#2577
GPX4	Cell Signaling	#52455
FDX1/ADX	Abcam	ab108257
GSDMD	Cell Signaling	#39754
MLKL	Cell Signaling	#14993
p-MLKL	Cell Signaling	#91689
β-actin	Sigma-Aldrich	A3854
α-tubulin	Cell Signaling	#2144

### MTT Assay

Cells were seeded in 96-well plates at 1–3 × 10^4^ cells/well density. After treatment, samples were incubated with MTT (0.5 mg/mL, Sigma-Aldrich, catalog no. M5655) for 3.5 h. Formazan crystals were solubilized in isopropanol and read in a spectrophotometer at 570 nm absorbance.

### Trypan Blue Exclusion Assay

Cells were seeded in 24-well plates at densities of 1–3 × 10^4^ cells/well and treated with increasing concentrations of the copper­(II) complex for 24 h. Samples were stained with trypan blue solution (4%) at a 1:1 dilution, and counted using a Neubauer chamber, with blue cells considered nonviable and unstained cells considered viable.

### Clonogenic Formation Assay

Following the protocol described previously,^71^ cells were seeded in 12-well plates at a density of 1–2 × 10^3^ cells/well. Cells were treated with the binuclear copper­(II) complex every 3–4 days and maintained in culture for 10–15 days. Upon reaching 80–100% confluence, samples were fixed with 6% formaldehyde and stained with 1% crystal violet for 30 min. Wells were scanned at 600 dpi and analyzed using Fiji 2.16 software.^72^ Data were expressed as mean ± standard error of the mean (n = 3) and normalized to 100% relative to the untreated control mean for well area occupancy.

### Wound-Healing Assay

BRAFi-resistant SKMEL28 melanoma cells were seeded in 24-well plates in triplicate at a density of 1 × 10^5^ cells/well. After cells reached 80–90% confluence, a scratch/gap was created using a p200 pipet tip. Cells were treated with binuclear copper­(II) complex diluted in DMEM supplemented with 1% fetal bovine serum to avoid proliferation. To monitor the closure of the central wound, at least two points per well were photographed at time 0 (immediately after the wound creation), 24 h, and 48 h. Images were analyzed with Fiji 2.16 following protocol described elsewhere.^73^

### Boyden-Chamber Invasion Assay

Cells were seeded in the upper chamber containing serum-free DMEM over the porous membrane (8 μm, Corning, catalog no. 353097) coated with Matrigel (1:6 Matrigel/DMEM, Corning, catalog no. 354234), while the lower chamber contained DMEM supplemented with 10% FBS. The copper­(II) complex was added to both chambers, and the ability of the cells to traverse the Matrigel-coated membrane and adhere to its lower surface was evaluated. Cells on the lower surface were stained with 0.5% crystal violet (in methanol and water, 1:1) for 10 min. For quantification, at least four random fields were counted using an inverted microscope with a 10× objective.

### Reconstructed Human Skin (RHS)

Artificial skin reconstruction followed the RHS protocol described by our group.^70^ To produce dermal equivalents, a type I collagen matrix (Corning, catalog no. 354236) was prepared to support the seeding of 1.5 × 10^5^ fibroblasts/well in a 24-well plate. After dermal equivalent polymerization, keratinocytes and melanocytes were seeded at densities of 2.5 × 10^5^ and 0.8 × 10^4^ cells/well, respectively.

For melanoma groups, either SKMEL28 parental or BRAFi-resistant cells were added at a density of 5 × 10^5^ cells/well. After complete collagen I gel polymerization (overnight in a 37 °C incubator with 5% CO_2_), skin samples were transferred onto a stainless-steel mesh in a 6-well plate, leaving the skin at the air–liquid interface. The lower part was in contact with RAFT culture medium consisting of 67.5% DMEM, 22.5% Ham’s F-12, 10% fetal bovine serum, 5 μg/mL apo-transferrin (Sigma-Aldrich, catalog no. T-1147), 5 μg/mL insulin (Sigma-Aldrich, catalog no. I-1882), 0.4 μg/mL hydrocortisone hemisuccinate-21 (Sigma-Aldrich, catalog no. H-4881), 0.5 ng/mL EGF (human epidermal growth factor, Gibco/Invitrogen, catalog no. 13247–010), and 0.1 nM cholera toxin (Sigma-Aldrich, catalog no. 3012). RAFT medium was renewed every 48–72 h. Following 10 days of culture and skin tissue layer stratification, RHS samples were treated three times for 24-h periods with 30 μM of the binuclear copper­(II) complex, and samples were collected on the 13th day. Treated and nontreated samples were fixed in 4% paraformaldehyde for 4 h and 70% alcohol for 24 h prior for the histological process. RHS samples were stained with hematoxylin/eosin (H&E) for histological analysis.

### Reactive Oxygen Species (ROS) Production Assessment

Cells were seeded in black 96-well plates at a density of 5 × 10^4^ cells/well and treated with the binuclear copper­(II) complex, doxorubicin (5 μM), H_2_O_2_ (200 mM), or a coexposure throughout the treatment with N-acetylcysteine (NAC, 1 mM) (Sigma-Aldrich, catalog no. A7250) for 30 min. After treatment, cells were washed with PBS and incubated with PBS supplemented with 1 mg/mL glucose and dihydrorhodamine 123 (DHR 123, Thermo Fisher Scientific, catalog no. D23806) at 20 μM for 3 h (180 min). Fluorescence intensity readings were taken every 10 min using a spectrophotometer with excitation at 485 nm and emission at 528 nm.

### Flow Cytometry

Cell lines were seeded in 6-well plates and incubated with binuclear copper­(II) complex. Following incubation, samples were detached, washed twice with PBS, and stained according to each reagent and manufacturer’s protocols previously described by our group.^74^ Samples were analyzed by flow cytometry considering at least 10,000 events representing each cell.

### Cell Cycle Analysis

After 24-h treatment with binuclear copper­(II) complex, cells were resuspended in membrane permeabilization buffer (phosphate buffer, 4 mg/mL RNase A, 0.1% Triton X-100) containing propidium iodide (5 μg/mL, Life Technologies, catalog no. 03566) and incubated for 30 min at room temperature in the dark. The emitted fluorescence was measured by flow cytometry and analyzed based on intensity meaning DNA content.

### Mitochondrial Membrane Integrity Assessment (ΔΨm)

Cells were treated with binuclear copper­(II) complex for 24-h period and stained with MitoProbe TMRM Kit (20 nM, Thermo Fisher Scientific, catalog no. M20036) according to the manufacturer’s instructions. After 30 min of staining at room temperature in the dark, cells were analyzed by flow cytometry based on fluorescence intensity. Carbonyl cyanide *m*-chlorophenyl hydrazone (CCCP, 50 μM) was used as a positive control for mitochondrial membrane depolarization.

### Annexin V and Propidium Iodide (PI) Cell Death Assessment

After 24-h treatment with binuclear copper­(II) complex and/or pre- 1-h co- treatment NAC (10 mM), ferrostatin-1 (5 μM, MedChemExpress, catalog no. HY-100579), necrostatin-1 (30 μM, MedChemExpress, catalog no. HY-15760), and bafilomycin A1 (0.01 μM, MedChemExpress, catalog no. HY-100558). Cells were subjected to staining with the FITC Annexin V Apoptosis Detection Kit II (BD Biosciences, catalog no. 556570), according to the manufacturer’s instructions. After 15 min of staining at room temperature in the dark, cells were analyzed by flow cytometry.

### Quantitative Real-Time PCR Array

Parental and drug-resistant melanoma cells were incubated with either vehicle or the test compound at a final concentration of 20 μM for 24 h. Total RNA was extracted using the TRIzol reagent (Thermo Fisher Scientific, catalog no. 15596026). Subsequently, 1 μg of RNA was reverse transcribed into cDNA using the High-Capacity cDNA Reverse Transcription Kit (Thermo Fisher Scientific). Quantitative real-time PCR (qPCR) was performed with the QuantStudio 3 Real-Time PCR System (Thermo Fisher Scientific), employing the SybrGreen detection method (Thermo Fisher Scientific, catalog no. 4367659), and gene-specific primers listed in Table 4. HPRT1 and ACTB were used as internal reference genes. Gene expression was quantified using the 2^–ΔΔCT^ method. A no-template control (NTC) was included for each primer pair to ensure specificity.

**Table 4:** Sequences and Concentrations of Primers Used in the Quantitative PCR Reaction and Cellular Processes Related to the Analyzed Genes of Interest^a^

Gene	Sequence	Concentration	Cellular process
*ATG5*	FW: GGGCCATCAATCGGAAAC	300 nM	autophagy
	RV: AGCCACAGGACGAAACAG		
*BAK1*	FW: TGACTTCACCAAGATTGCCA	300 nM	apoptosis
	RV: AGTCAGGCCATGCTGGTAGAC		
*BBC3*	FW: GACCTCAACGCACAGTACGAG	300 nM	DNA damage/apoptosis
	RV: AGGAGTCCCATGATGAGATTG		
*BCL2L11*	FW: ATGTCTGACTCTGACTCTCG	300 nM	apoptosis
	RV: CCTTGTGGCTCTGTCTGTAG		
*BIRC5*	FW: GCCCAGTTCTTCTGCTTC	300 nM	apoptosis
	RV: GCACTTTCTCCGCAGTTTCCTC		
*CCNA2*	FW: GCCTTTCATTTAGCACTCTACA	300 nM	cell cycle
	RV: CAGGGTATATCCAGTCTTTCG		
*CCNB1*	FW: GTCTCCATTATTGATCGGTTCATG	300 nM	cell cycle
	RV: CCAATTTCTGGAGGGTACATTTCT		
*CCND1*	FW: CTCGGTGTCCTACTTCAAATG	300 nM	cell cycle
	RV: AGCGGTCCAGGTAGTTCAT		
*CCNE1*	FW: TATATGGCGACACAAGAAAATG	300 nM	cell cycle
	RV: GTGCAACTTTGGAGGATAGA		
*CDKN1A*	FW: TGTCACTGTCTTGTACCCTTGT	300 nM	cell cycle
	RV: GCCGGCGTTTGGAGTGGTAG		
*CDKN1B*	FW: ACTCTGAGGACACGCATTTGGT	300 nM	cell cycle
	RV: TCTGTTCTGTTGGCTCTTTTGTT		
*GADD45A*	FW: AAGGATGGATAAGGTGGGG	300 nM	DNA damage
	RV: CTGGATCAGGGTGAAGTGG		
*MAP1LC3B*	FW: AAGGCGCTTACAGCTCAATG	150 nM	autophagy
	RV: CTGGGAGGCATAGACCATGT		
*MCL1*	FW: GTAATAACACCAGTACGGACGG	300 nM	apoptosis
	RV: TCCCGAAGGTACCGAGAGAT		
*PMAIP1*	FW: CGCGCAAGAACGCTCAACC	300 nM	DNA damage/apoptosis
	RV: CACACTCGACTTCCAGCTCTGCT		
*SQSTM1/p62*	FW: TGAGGAACAGATGGAGTCGGATAA	300 nM	autophagy
	RV: GGGACTGGAGTTCACCTGTAGACG		
*HPRT1*	FW: GAACGTCTTGCTCGAGATGTGA	150 nM	reference gene
	RV: TCCAGCAGGTCAGCAAAGAAT		
*ACTB*	FW: AGGCCAACCGCGAGAAG	150 nM	reference gene
	RV: ACAGCCTGGATAGCAACGTACA		

a Abbreviations: FW, forward; RV, reverse. Gene names conform to the HUGO nomenclature for genes and proteins.

### Statistical Analysis

Quantitative data are expressed as mean ± standard error of the mean (SEM) from at least three independent experiments (*n* ≥ 3). Statistical significance (*p* < 0.05) was assessed using Student’s *t* test or One-way/Two-way analysis of variance (ANOVA).

## Supplementary Material



## Notes

MTT dose–response curves from the cytotoxicity screening of mononuclear [Cu­(II)­L], dacarbazine, and cisplatin (Figure S1); MTT assay results for the combination treatment of binuclear [Cu­(II)­L] and vemurafenib (Figure S2); micrographs displaying the morphology of melanoma cells following treatment with binuclear [Cu­(II)­L] (Figure S3); and the evaluation of cell death via Annexin V/PI staining upon [Cu­(II)­L] cotreatment with the caspase inhibitor z-VAD-fmk, alongside Western blot of caspases 3 and 9 after treatment with binuclear [Cu­(II)­L] (Figure S4) (PDF)

## Abbreviations

-P: Parental
-R: BRAFi-resistant
ACTB: Actin Beta
ATG5: Autophagy related 5
AXL: AXL receptor tyrosine kinase
BAK1: BCL2 antagonist/killer 1
BBC3: BCL2 binding component 3
BCL2L11: BCL2 like 11
BIRC5: Baculoviral IAP repeat containing 5
BRAF: B-Raf proto-oncogene
BRAFi: BRAF inhibitor
CCCP: Carbonyl cyanide *m*-chlorophenyl hydrazone
CCNA2: Cyclin A2
CCNB1: Cyclin B1
CCNE: Cyclin E
CDKN1A (p21): Cyclin dependent kinase inhibitor 1A
CDKN1B (p27): Cyclin dependent kinase inhibitor 1B
DHR 123: Dihydrorhodamine 123
DMEM: Dulbecco’s Modified Eagle Medium
DNA: Deoxyribonucleic acid
EGF: Epidermal growth factor
FDX1: Ferredoxin 1
GADD45A: Growth arrest and DNA damage inducible alpha
GPX4: Glutathione peroxidase 4
GSDMD: Gasdermin D
H&E: Hematoxylin and eosin
H2AX (p-H2AX): H2A.X variant histone
HPRT1: Hypoxanthine phosphoribosyltransferase 1
IC_50_: Half maximal inhibitory concentration
LC3/LC3B/MAP1LC3B: Microtubule associated protein 1 light chain 3 beta
MAPK: Mitogen-activated protein kinase
MCL1: Myeloid cell leukemia 1
MITF: Melanocyte inducing transcription factor
MLKL (p-MLKL): Mixed lineage kinase domain like pseudokinase
MTT: 3-(4,5-dimethylthiazol-2-yl)-2,5-diphenyltetrazolium bromide
NAC: N-acetylcysteine
SQSTM1 (p62): Sequestosome 1
PARP1: Poly­(ADP-ribose) polymerase 1
PI: Propidium iodide
PMAIP1: Phorbol-12-myristate-13-acetate-induced protein 1
qPCR: Quantitative real-time polymerase chain reaction
RHS: Reconstructed human skin
ROS: Reactive oxygen species
SDS: Sodium dodecyl sulfate
TMRM: Tetramethylrhodamine methyl ester
