# Supplementary material for: Antiproliferation and Anti-invasion Activity of Binuclear Copper(II) Complex in BRAFi-Resistant Melanoma Cells
Source: ACS Pharmacol Transl Sci. 2026 Aug 10;9(9):2454–68. doi: 10.1021/acsptsci.6c00033 (PMC13632889; doi:10.1021/acsptsci.6c00033)
Supplement: Supplementary file 1 [file pt6c00033_si_001.pdf]

SUPPORTING INFORMATION

ANTIPROLIFERATION AND ANTI-INVASION ACTIVITY OF BINUCLEAR COPPER(II) COMPLEX IN BRAFi-RESISTANT MELANOMA CELLS

Manoel Oliveira de Moraes Junior[1], *Larissa Anastácio da Costa Carvalho*[1], *Yasmin Catherine da Paz Mendonça*[1], *Renaira Oliveira da Silva*[1], *Cleia Justino Nunes*[2], *Keli Lima*[3], *João Agostinho Machado-Neto*[3], *Tiago Rodrigues*[4], *Vito Rebecca*[5], *Corine Bertolotto*[6], *Ana Maria da Costa Ferreira*[2], *Silvya Stuchi Maria-Engler*[1,*]

1 Department of Clinical and Toxicological Analyses, School of Pharmaceutical Sciences, University of São Paulo, São Paulo, SP 05508-000, Brazil.

2 Department of Fundamental Chemistry, Chemistry Institute, University of São Paulo, São Paulo, SP 05508-000, Brazil.

3 Department of Pharmacology, Institute of Biomedical Sciences, University of São Paulo, São Paulo, SP 05508-000, Brazil.

4 Center for Natural and Human Sciences, Federal University of ABC, Santo André, SP 09210-580, Brazil.

5 Department of Biochemistry and Molecular Biology, Bloomberg School of Public Health, Johns Hopkins University, Baltimore, MD 21205, USA.

6 Centre Méditerranéen de Médecine Moléculaire, Université Côte d'Azur, Nice, PACA 06103, France.

*Corresponding author email: silvya@usp.br.

Total Pages: S1-S5; Total Figures: S1-S4

TABLE OF CONTENTS

Figure S1: Cytotoxicity screening of mononuclear [Cu(II)L], dacarbazine, and cisplatin in parental and resistant melanoma cell lines..S3

Figure S2: Parental melanoma cells do not benefit from binuclear [Cu(II)L] combination with vemurafenib (BRAFi) in 72-hour treatment…………………………………………………...S3

Figure S3: Morphology of melanoma cells after 24-hour treatment with binuclear [Cu(II)L]…...S4

Figure S4: Binuclear [Cu(II)L] effect does not depend on caspase activity……………………...S5

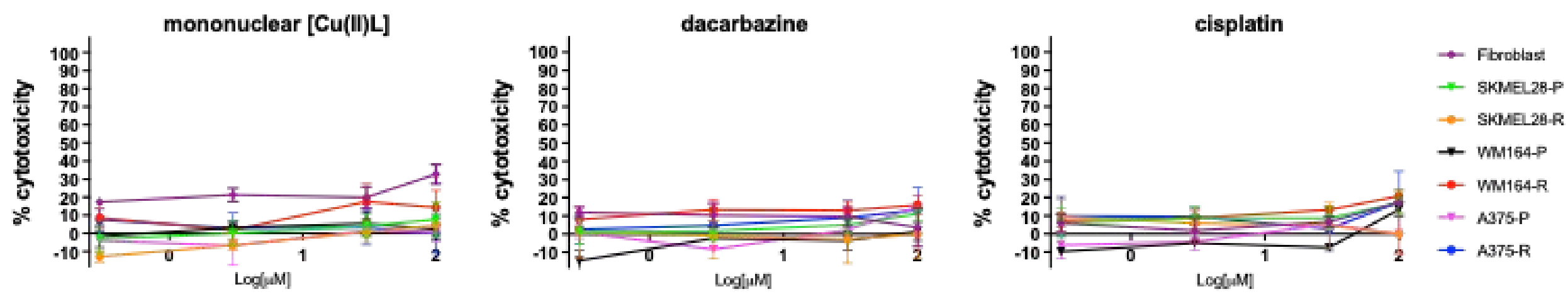

Figure S1: Cytotoxicity screening of mononuclear [Cu(II)L], dacarbazine, and cisplatin in parental and resistant melanoma cell lines. MTT assay following 24-hour of treatment. Results represent mean ± standart error of the mean (n = 3).

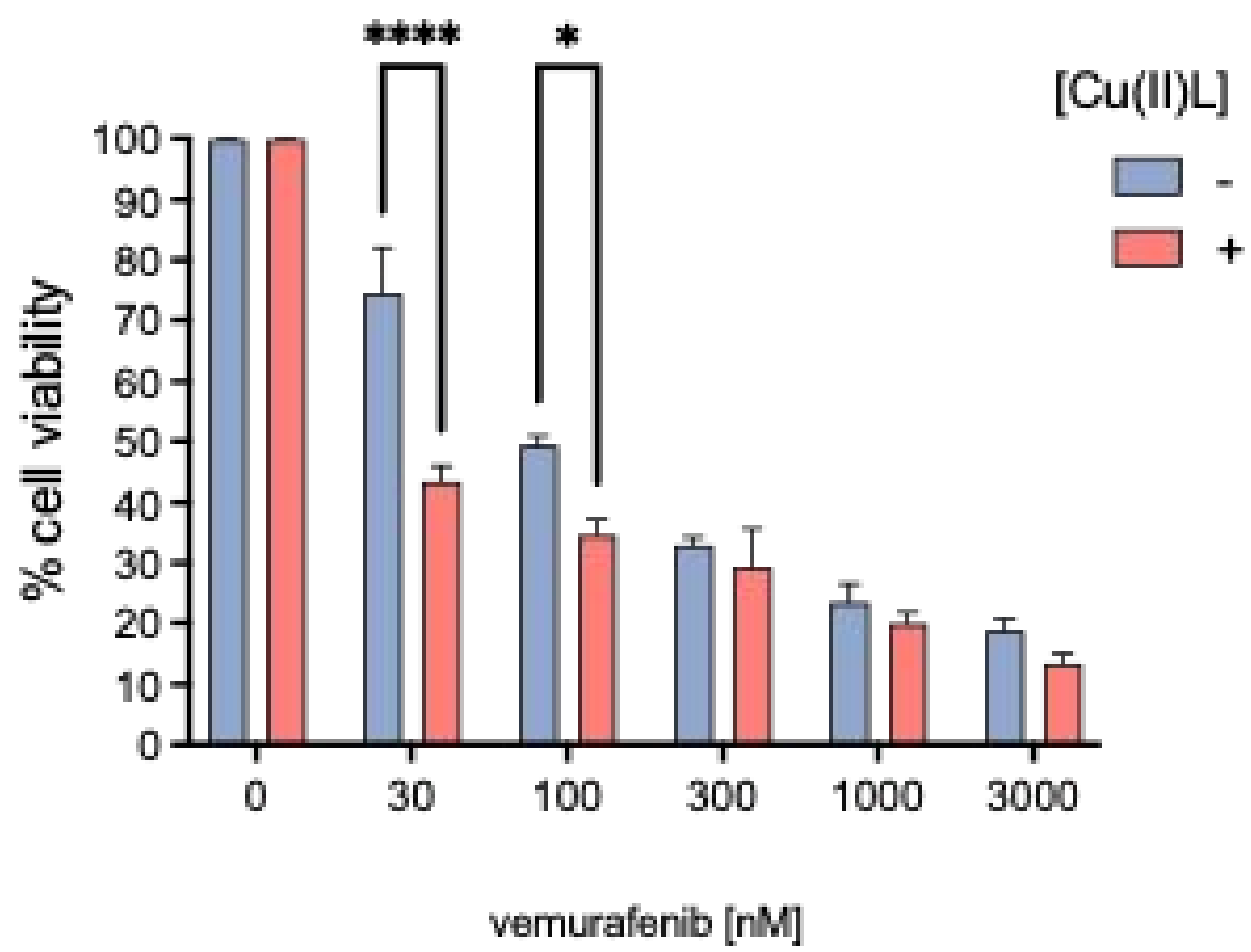

Figure S2: Parental melanoma cells do not benefit from binuclear [Cu(II)L] combination with vemurafenib (BRAFi) in 72-hour treatment. MTT assay bar graph shows SKMEL28-P co-treated with increasing concentration of vemurafenib (30-3000 nM) and binuclear [Cu(II)L] (7500 nM) Statistical data are expressed as mean ± standard error of the mean (n = 3).

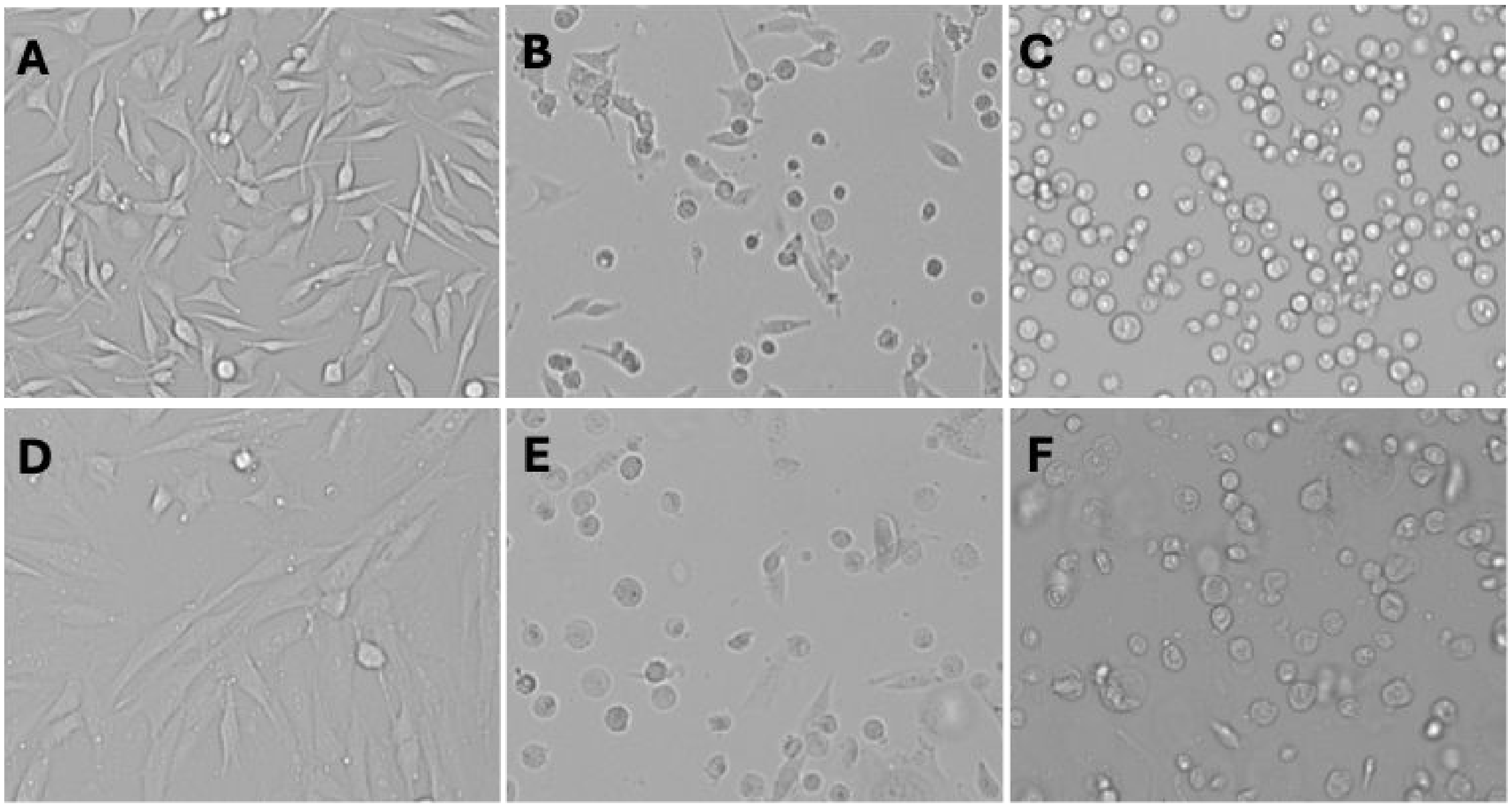

Figure S3: Morphology of melanoma cells after 24-hour treatment with binuclear [Cu(II)L]. (A) Untreated parental cells; (B) parental cells treated with 15 μM binuclear [Cu(II)L]; (C) parental cells treated with 30 μM binuclear [Cu(II)L]; (D) untreated resistant cells; (E) resistant cells treated with 15 μM binuclear [Cu(II)L]; (F) resistant cells treated with 30 μM binuclear [Cu(II)L]. Images acquired after 24-hour incubation in SKMEL28 cells. Inverted microscopy images at 10 × magnification.

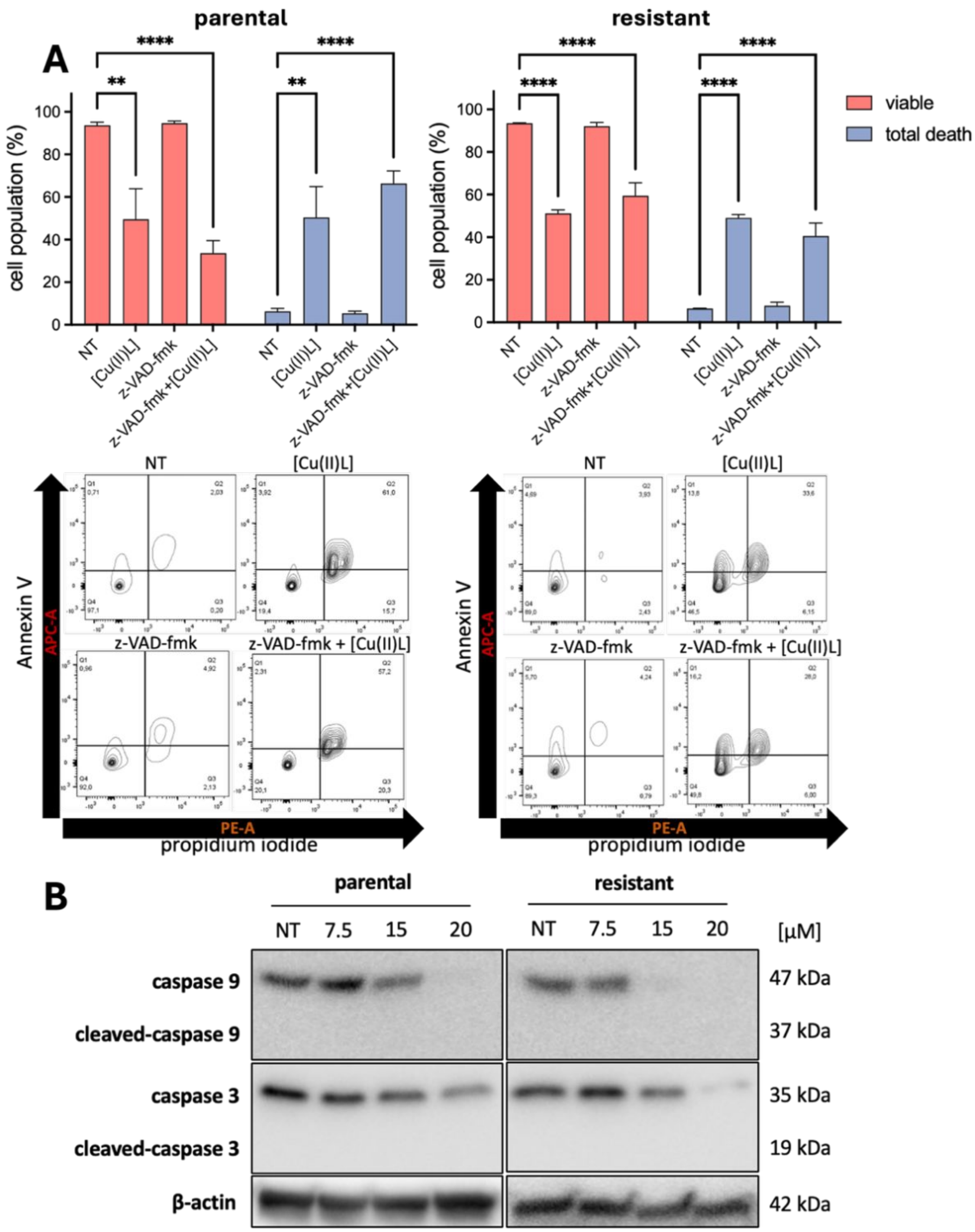

Figure S4: Binuclear [Cu(II)L] effect does not depend on caspase activity. (A) AnnexinV and PI cell death evaluation upon [Cu(II)L] (20 µM) co-treatment with caspase-inhibitor z-VAD-fmk (20 µM); (B) western blot evaluation on caspase 3 and caspase 9 cleavage. (A-B) tested with 24-hour incubation in SKMEL28 cells. **$p<0.01$ and ****$p<0.0001$ compared to NT control; NT = non-treated. Two-way ANOVA was applied to (A).
